# Vibration Anatomy and Damage Detection in Power Transmission Towers with Limited Sensors

**DOI:** 10.3390/s20061731

**Published:** 2020-03-20

**Authors:** R. Karami-Mohammadi, M. Mirtaheri, M. Salkhordeh, M. A. Hariri-Ardebili

**Affiliations:** 1 Hybrid Simulation Laboratory, Faculty of Civil Engineering, K. N. Toosi University of Technology, Tehran 19967-15433, Iran; rkarami@kntu.ac.ir (R.K.-M.); mmirtaheri@kntu.ac.ir (M.M.); m-salkhordeh@email.kntu.ac.ir (M.S.); 2 University of Maryland, College Park, MD 20742, USA; 3 Department of Civil Environmental and Architectural Engineering, University of Colorado, Boulder, CO 80303, USA

**Keywords:** power transmission tower, signal processing, wavelet transform, damage detection, optimal sensor location

## Abstract

This study presents a technique to identify the vibration characteristics in power transmission towers and to detect the potential structural damages. This method is based on the curvature of the mode shapes coupled with a continuous wavelet transform. The elaborated numerical method is based on signal processing of the output that resulted from ambient vibration. This technique benefits from a limited number of sensors, which makes it a cost-effective approach compared to others. The optimal spatial location for these sensors is obtained by the minimization of the non-diagonal entries in the modal assurance criterion (MAC) matrix. The Hilbert–Huang transform was also used to identify the dynamic anatomy of the structure. In order to simulate the realistic condition of the measured structural response in the field condition, a 10% noise is added to the response of the numerical model. Four damage scenarios were considered, and the potential damages were identified using wavelet transform on the difference of mode shapes curvature in the intact and damaged towers. Results show a promising accuracy considering the small number of applied sensors. This study proposes a low-cost and feasible technique for structural health monitoring.

## 1. Introduction

**Figure 1:**
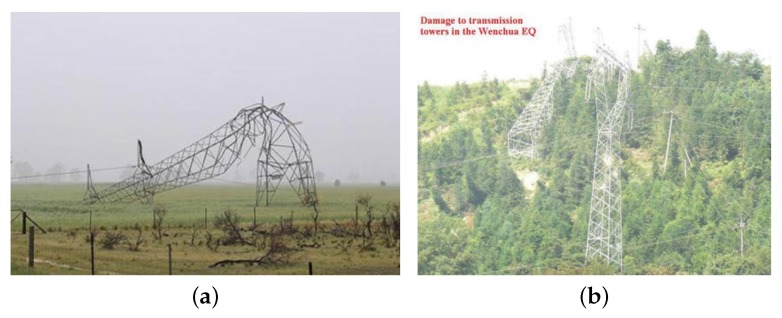
Potential failures in power transmission towers [2,3]. (**a**) Wind-induced failure; (**b**) Earthquake- induced failure.

Continuous health monitoring of transmission networks is a vital task to prevent sudden failure in power transmission lines. The substations and transmission lines transmit the electricity production of the power plants through various physical, atmospheric, and geographical conditions to the consumption centers. Performance of the transmission lines is sometimes affected negatively by some factors, which may disrupt the power distribution network. Such incidents may cause irreparable damages. The cost of repairing the power transmission lines is very high, at about one million dollars per kilometer [1]. Such towers are not only damageable against the wind loads, the world-wide experiences show their vulnerability subjected to earthquake ground motions and storm events, as well. Figure 1 illustrates the potential failures under wind and earthquake loading [2,3].

Despite several studies that have studied the vibration characteristics of towers under earthquake and wind loads [4], the health monitoring of these structures has received limited attention, especially from industrial practices. Indeed, structural health monitoring (SHM) techniques are key methods in the risk-based safety assessment of such tower-line systems. In recent years, SHM techniques have been extended both from theoretical and applied aspects [5].

### Objectives and Contributions

In the present study, a vibration-based technique is presented to identify the spatial location of existing damage in the power transmission towers. An important advantage of this method is that it is a cost-effective method using limited number of sensors compared to other damage detection techniques. This method is based on post-processing the structural responses under ambient vibration (as opposed to expensive, dangerous, and more complex forced vibration techniques).

An existing tower was used as case study. First, it was analyzed using a finite element model developed in the SAP2000 software, and the vibration information (i.e., fundamental frequencies and mode shapes) were extracted. Next, they were verified through conducting a field measurement test. Finally, in order to simulate a more realistic field condition, a 10% noise was added to the structural responses. To optimize the number of required sensors, a method based on minimizing the non-diagonal entries of modal assurance criterion (MAC) matrix was used. Free vibration response of the structure for all the sensors was obtained using the natural excitation technique (NexT) [6]. Subsequently, the Hilbert–Huang transform and continuous wavelet transform (CWT) were used to extract the modal parameters. Last but not least, the structural damage was identified by implementing the CWT on the difference of the mode shape curvatures of the intact and damaged structure.

The structure of the paper is as follows: Section 2 provides a comprehensive overview on the current literature. Section 3 presents the proposed methodology and corresponding theoretical background. Section 4 describes the case study tower, the modeling procedure, validation of numerical model, and the parametric study. Section 5 proposes an optimization algorithm to find the minimum number of required sensors to be located in a tower. Section 6 describes the procedure implemented to identify the dynamic characteristics of the tower. Moreover, the noise effect on the results is investigated in this section. The proposed damage detection method is clarified in Section 7. Finally, the major outcomes are summarized in Section 8.

## 2. A State-of-the-Art Literature Review

The first section of literature review, Section 2.1, provides a general overview on the recent advances in structural damage detection and SHM. It is followed by a series of detailed literature reviews on damage detection in power transmission towers, which is in Section 2.2.

### 2.1. Structural Damage Detection

Montejo [7] compared different vibration-based damage detection techniques to identify the damage occurrence in the structures subjected to random excitation. The authors reported that the continuous wavelet transform (CWT)-based technique is more effective compared to the uncovering of spikes in the high frequency component of the structural response obtained via discrete wavelet transforms (DWT), Hilbert–Huang transforms, or high pass filtering. Liu et al. [8] presented a novel method based on stationary wavelet transform (SWT) to identify the length and the location of cracks occurred in a cantilever beam. Garcia-Palencia et al. [9] presented a method based on the frequency response functions (FRFs) and model updating algorithm. This method was validated using data obtained from the University of Central Florida’s benchmark structure.

Ditommaso et al. [10] proposed a methodology for the damage localization of framed structures that were subject to strong motion earthquakes based on monitoring the modal curvature variation in the natural frequencies. They have verified the proposed method using finite element modeling, as well as multiple experimental tests. Zhang et al. [11] proposed some damage indices based on the macro-strain modal shapes to identify the potential damage in steel stringer bridges. Yazdanpanah1a and Seyedpoor [12] proposed a novel indicator based on mode shape data for damage detection in beam-like structures. The method is based on three factors: the mode shape, the slope of the mode shape, and the curvature of the mode shape.

Ghiasi et al. [13] presented a novel damage detection approach based on least square support vector machine (LS-SVM). They proposed a new kernel function based on thin plate spline Littlewood–Paley wavelet kernel function. Lv et al. [14] proposed a combined method based on both variational mode decomposition (VMD) and multi-kernel support vector machine (MK-SVM) optimized by an immune genetic algorithm (IGA) for damage detection in mechanical systems. Cha et al. [15] proposed a method based on image processing to detect the damages in civil infrastructures. They employed features obtained from image processing along with the convolutional neural networks, to perform damage detection in the structures. Zhao et al. [16] conducted a comparative study based on wavelet multi-resolution, wavelet packet energy, and fuzzy sets to identify the existing damage in beam-like structures.

Karami-Mohammadi et al. [17] proposed a combined method based on FRFs and principal component analysis (PCA) to identify the damage occurrence in the capacitive voltage transformer (CVT), an equipment of power transmission posts. Vahidi et al. [18] presented a model-updating-based method for damage detection in structures. They have minimized the difference between the modal response of the finite element model and the experimental one by updating the numerical model in several iterations. To achieve this goal, they have utilized particle swarm optimization (PSO), genetic algorithm (GA), and artificial bee colony (ABC) techniques. Chang et al. [19] proposed an artificial neural network (ANN)-based hybrid method by tracking the changes in natural frequencies and structural mode shape variations (as two indicators). They applied this method to multi-story buildings. Ghannadi and Kourehli [20] investigated the application of the moth flame optimization algorithm to detect the structural damage. They used the natural frequencies and MAC flexibility as damage indicators. Nguyen et al. [21] proposed a method based on both the transmissibility function and ANNs to identify the structural damage in bridges. They validated their method on an existing bridge in Taiwan.

### 2.2. Damage Detection of Power Transmission Equipment

On the other hand, there are only a few studies on the application of damage detection techniques in power transmission equipment. According to Qu et al. [22], power transmission towers are susceptible to sudden damages and instabilities which may lead to total collapse of the structure. Huang et al. [23] investigated the effect of foundation settlement on the variations in natural frequencies of the tower. Lam and Yang [24] proposed a method based on the Bayesian probabilistic approach and finite element model updating for damage detection in power transmission towers. Variations in modal parameters of towers were used as damage indicator. Yin et al. [25] utilized the dynamic reduction technique to identify the structural damage in the power transmission towers. In order to simulate the actual field condition, they added some noise to the acceleration response obtained from their numerical model.

Qu et al. [22] presented a two-stage method to identify the damage in the vertical elements of the power transmission towers. In this method, wavelet packet energy and modal strain energy of the structure were used as a damage indicator. Xu et al. [2] proposed a multi-stage procedure based on the covariance matrix of the dynamic response of the structure for damage detection in the connections of the power transmission towers. The main drawback of this procedure was the large number of applied sensors, which makes it expensive and impractical in a majority of cases.

## 3. Proposed Methodology and Underpinning Theories

### 3.1. Summary of the Proposed Method

**Figure 2:**
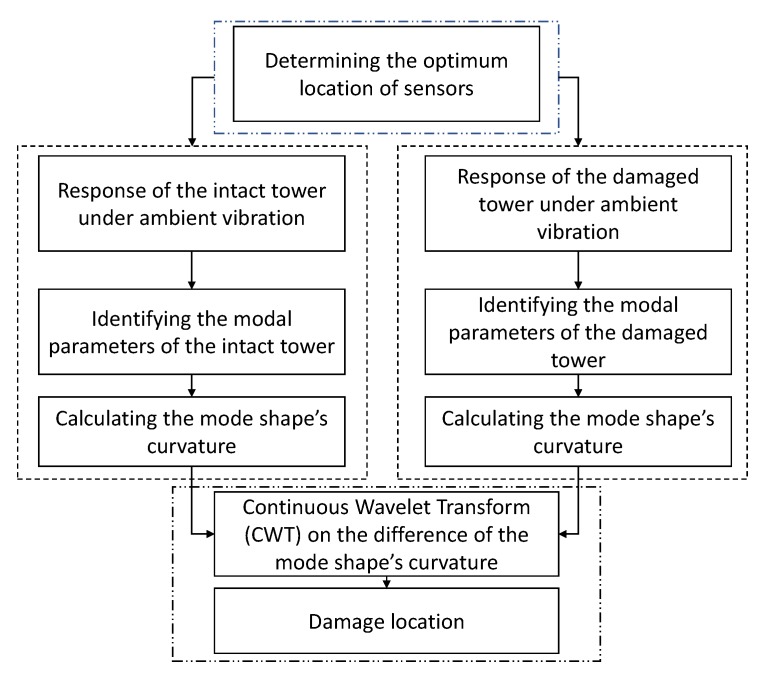
Main algorithm of the proposed method.

This study aims to implement a cost-effective procedure to identify the location of damage in the power transmission towers. The optimum number of sensors, as well as their location is determined by minimizing the non-diagonal entries of the MAC matrix. Subsequently, the modal parameters of the structure will be determined by processing the output signal of the tower under ambient vibration. As a result of structural damage, the vibration anatomy of the towers changes. The proposed method computes the differences between curvature of the mode shapes in the intact and damaged structures. Finally, by implementing the continuous Wavelet transform on residual of the curvature of the mode shapes, the damage location is determined. Figure 2 presents the main algorithm of the proposed method.

### 3.2. Theoretical Background of Continuous Wavelet Transform

Since the essence of the proposed procedure is founded on the concept of CWT, a brief theoretical background is presented in this section for those readers less familiar with this topic. The underpinning theory of wavelets is connected to continuous wavelet decomposition of $L^{2}$ functions [26,27].

If $\psi_{a,b}$, $a\in R\;\backslash \left\{0\right\}$, b∈R is are functions defined as the translations and re-scales of a single function $\psi \left(x\right)\in L^{2}\left(R\right)$:

(1) $$\psi_{a,b}\left(t\right)={\left|a\right|}^{-0.5}\psi \left(\frac{t-b}{a}\right)$$

where *a* is the scale parameter, *b* is the time location, and ${\left|a\right|}^{-0.5}$ is used to ensure that $\left|\left|\psi_{a,b}\right|\right|$ is independent of *a* and *b*. Also, ψ is called the wavelet function or the mother wavelet, and must satisfy the admissibility condition:

(2) $$C_{\psi }=\int_{-\infty }^{\infty }\frac{{\left|\hat{\psi }\left(\omega \right)\right|}^{2}}{\left|\omega \right|}d\omega <\infty$$

where ω is the frequency, and $\hat{\psi }\left(\omega \right)=\int_{R}\psi \left(x\right)\exp\left(-ix\omega \right)dx$ is the Fourier transform of ψ.

The CWT of the signal x(t) is defined as the inner product of the Hilbert space of $L^{2}$ norms as shown below.

(3) $$W_{b}\left(a\right)=<\psi_{a,b}\left(t\right),x\left(t\right)>={\left|a\right|}^{-0.5}\int x\left(t\right)\psi_{a,b}^{*}dt.$$

where the asterisk stands for complex conjugate, and the scale factors *a* and *b* vary continuously.

Wavelet functions are divided into two groups: orthogonal and non-orthogonal ones. In dyadic Discrete Wavelet Transform (DWT), as well as the wavelet packet transform, one should select the orthogonal wavelet function. On the other hand, using CWT, one can select either orthogonal or non-orthogonal wavelet functions. Some of the most well-known orthogonal wavelets are: Haar, Daubechies, Coiflets, Meyer, etc [28]. Further, Morlet, Mexican hat, and Difference of Gaussian (DOG) wavelets are some of the non-orthogonal functions. Applying a CWT to a signal, if the variations of the wavelet are similar to the variation of the mentioned signal, the corresponding wavelet coefficients become larger [29]. The following is a summary of several well-known wavelets [30]:

- Gaussian, Morlet, Mexican Hat, and Shannon wavelets are models in which the wavelet function, ψ, has an explicit expression. The scaling function does not exist for these wavelets, and thus, DWT, fast wavelet transform (FWT), and discrete reconstruction are unavailable. Analysis with these wavelets is limited to CWT.
- Meyer wavelet is an infinity regular wavelet. It does not have an explicit expression form, but the scaling function does exist, and using DWT is possible (FWT is still unavailable).
- Daubechies wavelets of order *N*, Symlet wavelets of order *N*, Coiflet wavelet of order *N*, and Haar wavelet are examples of orthogonal wavelets. They do not have an explicit expression for the wavelet function ψ (except for Daubechies wavelet of order one which is similar to Haar wavelet).

According to this classification, the mother wavelet to be operated in CWT may or may not take the orthogonal wavelet form. Any signal that satisfies the admissibility condition can be used as a mother wavelet. In this study, two different mother wavelets were adapted, i.e., Morlet and Dibucci. The former one was used to identify the modal parameters of a tower with the following form:

(4) $$\psi \left(t\right)=\exp\left(-\frac{1}{2}\beta^{2}t^{2}\right)\cdot \cos\left(\pi t\right)$$

where *t* is time and β controls the shape of the basic wavelet.

By introducing two new variables: *a* as dilation and *b* as translation, a son wavelet can be written as:

(5) $$\psi_{a,b}\left(t\right)=\exp\left(-\frac{\beta^{2}{\left(t-b\right)}^{2}}{a^{2}}\right)\cdot \cos\left(\frac{\pi (t-b)}{a}\right)$$

**Figure 3:**
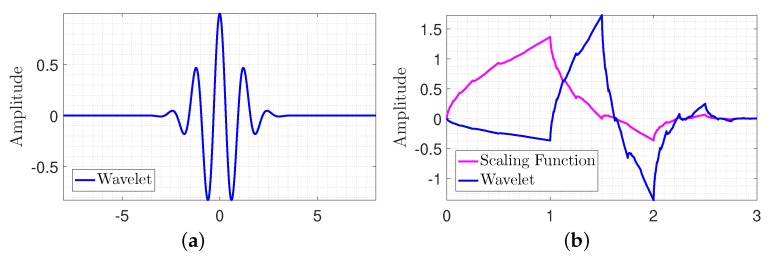
Visualization of wavelet functions. (**a**) Morlet wavelet; (**b**) Daubechies wavelet.

Clearly, it is a cosine signal that decays on both sides by the exponential term. For a digital signal, the sampling rate follows the Nyquist sampling theory; generally the sampling rate can be considered high enough. Then, it will have enough time resolution if the translation unit is equal to the sampling period. It is notable that in Morlet wavelet, parameter β balances the time and frequency resolution. Frequency resolution will increase by decreasing β value. Conversely, time resolution will increase by increasing β. When β yields to zero, the Morlet wavelet becomes a cosine function which has the best frequency resolution. Furthermore, when β tends to infinity, the Morlet wavelet converts to a Dirac function, which has the best time resolution. Figure 3a shows the shape of a Morlet wavelet [31,32].

Daubechies wavelets are defined by calculating the running averages and differences via scalar products with scaling signals and wavelets. Since the Daubechies wavelets use overlapping windows, the high frequency coefficient spectrum reflects all high frequency changes. It can be used as a proper wavelet to detect the high frequency jumps that occur because of damage in a response signal [33]. Therefore, the Daubechies mother wavelet was used to identify the structural damage occurred in the tower. Figure 3b shows Daubechies wavelet with two vanishing moments and the corresponding scaling function.

### 3.3. Theoretical Background of Cubic Spline

The cubic spline interpolation was introduced as an engineering tool used to draw smooth curves through a number of points. The cubic spline contains a series of weights attached to a flat surface at the points to be connected. A flexible strip is then bent across each of these weights, approaching a delicately smooth curve. The basic idea of the cubic spline was based on fitting a piece-wise function in the following form:

(6) $$S\left(x\right)=\left\{\begin{array}{cc} s_{1}\left(x\right) & \;\mathrm{if}\;x_{1}\leq x<x_{2}\\ s_{2}\left(x\right) & \;\mathrm{if}\;x_{2}\leq x<x_{3}\\ \; & \;...\\ s_{n-1}\left(x\right) & \;\mathrm{if}\;x_{n-1}\leq x<x_{n} \end{array}\right.$$

where $s_{i}$ is a 3rd-degree polynomial function.

A cubic spline needs to satisfy the following stipulations: (1) The piece-wise function S(x) interpolates all data points, and (2) S(x), $S^{\prime }\left(x\right)$ and $S^{\prime \prime }\left(x\right)$ should be continuous in $\left[x_{1},x_{n}\right]$. The cubic spline operation can be used to determine the rate of changes or cumulative change over an interval. In this study, since a small number of sensors are used for damage detection, the cubic spline operation generates a smooth curve of mode shape [34]. Consequently, it helps to derive the curvature of the mode shapes.

## 4. Case Study Tower

### 4.1. Tower Properties and Type of Conductors

**Figure 4:**
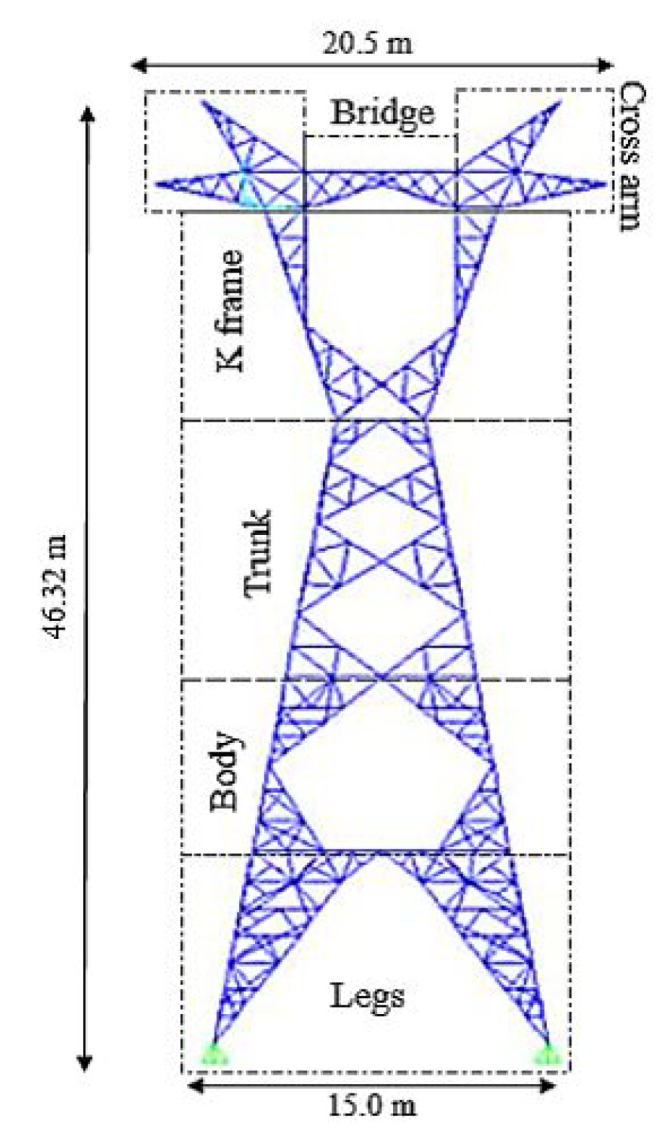
Geometry and dimensions of the case study tower.

The case study structure was a representative tower out of many, which is installed in the 400 kV line of Bandar Abbas-Sirjan route. This tower was known as S1KL in the power industry. The height of the tower was 46.32 m, see Figure 4. The distance between two adjacent legs was 15 m, and the dimension of its maximum cross section was 20.5 m (as mentioned in Section 1), this structure was marked as a middle-line tower which had a 400 m distance from adjacent towers. It is noteworthy that two adjacent towers were connected by conductors, which had a maximum sag about 17 m. Figure 4 shows the geometry of the tower. Furthermore, the conductors used for power transmission were pf a three-wire Curlew Bundle type.

### 4.2. Numerical Modeling

**Figure 5:**
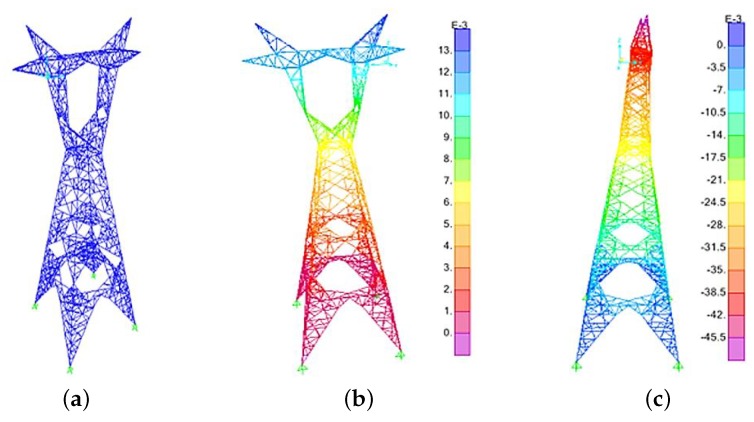
Numerical model and anatomy of vibration. (**a**) Finite element model; (**b**) First mode in latitudinal direction; (**c**) First mode in longitudinal direction.

The tower was modeled in the SAP2000 finite element software, see Figure 5a. According to the technical manual of the tower, the legs and the body of the tower were both made of frame elements. The rest of the tower including Trunk, K-frame, bridge, and cross-arms were made of truss elements. It should be mentioned that all elements were made of St37 steel. These elements were in the form of L45×3 and L120×10. Moreover, the tower contained a total of 1978 members and 852 connecting points. The total weight of the cables through effective length of the span (i.e., 400 m) was applied to a single tower through six points representing the location of the porcelains at which the cables were connected. Subsequently, the numerical modal analysis of the structure was carried out, and the fundamental frequencies of the tower in both perpendicular directions were extracted. Figure 5b,c shows the first mode shape of the tower in latitudinal and longitudinal directions, respectively.

### 4.3. Verification

**Figure 6:**
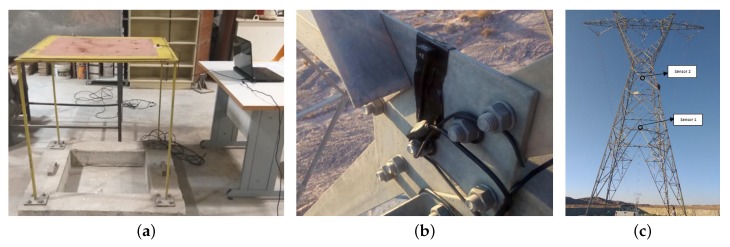
Verification of numerical modeling with experimental test. (**a**) Sensor calibration; (**b**) Sensor placement; (**c**) Sensors in tower.

The results of the numerical model were further verified by an ambient vibration test of the real tower. The accelerometers used in this experiment were Novinpardaz sensors with a sensitivity of 0.01 g. These sensors were first calibrated by measuring the natural frequency of the benchmark frame as shown in Figure 6a. The actual natural frequency of the benchmark frame was already reported in several studies to be 4.12 Hz [35]. The corresponding results of the accelerometer used in this study show a frequency of 4.07 Hz (i.e., only 1% error). Followed by this verification, the research team carefully prepared the test locations, as well as other equipment. Figure 6b,c shows the actual tower and the location of sensors. The results of the first two fundamental natural frequencies in each direction along with those obtained from the numerical model are shown in Table 1. Although there was an acceptable consistency between numerical natural frequencies and the corresponding experimental measurement, any differences can be attributed to the uncertainties in the properties of the modeled tower, such as mass and material uncertainty. Another source of uncertainty did exist in the modeling of interface between matching parts of the tower [36]. Moreover, there was a natural noise on the recorded response by sensors, which may affect the identified results. The verified numerical model was used as a base model for all subsequent damage detection analyses.

### 4.4. Parametric Study

**Figure 7:**
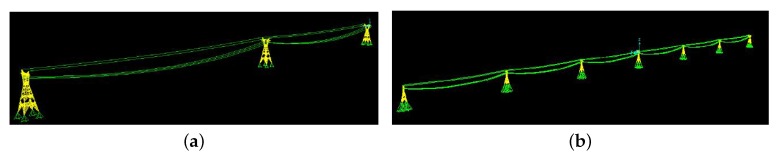
Interaction among towers. (**a**) Tower-line with three towers; (**b**) Tower-line with seven towers.

A parametric study was conducted to investigate the effect of the interaction among multiple towers in-line. For this purpose, the fundamental frequency of a single tower was compared with a series of towers (i.e., three, five, and seven towers), see Figure 7. The fundamental frequency of a single tower, tower-line with three, five, and seven towers are 3.25, 3.35, 3.33, and 3.32 Hz, respectively. As can be seen, the fundamental frequency of a single tower, when it is modeled considering the mass of conductors instead of modeling adjacent towers, is so close to the real state. Furthermore, by increasing the number of towers, the natural frequency of the tower-line approaches the frequency of the single tower.

## 5. Sensors Location Optimization

**Figure 8:**
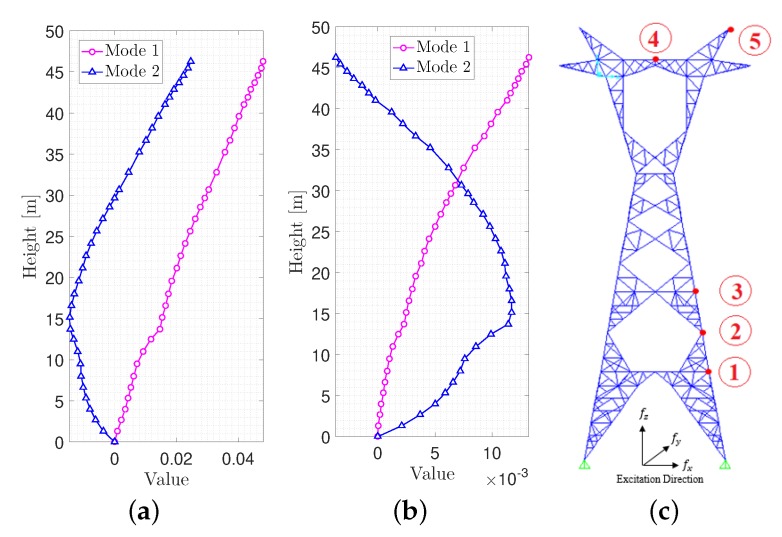
Mode shapes of the tower in 35 nodes along the height and optimal locations. (**a**) 1st and 2nd mode shapes; latitudinal direction; (**b**) 1st and 2nd mode shapes; longitudinal direction; (**c**) Optimal sensor combination.

Since the main objective of this paper was to minimize the number of sensors, the optimal placement of those instruments was very important to obtain the mode shapes of the tower. The team decided to use only five sensors in this research. The small number of sensors was selected based on two objectives: (1) economical constraints and (2) efficient coverage of the entire tower body in case of potential damage. The following steps were followed to find the optimal location:

- First, all the possible locations at which the sensors could be placed were identified (i.e., 35 nodes along the tower).
- Second, the modal analysis was conducted, and the mode shapes of the structure were obtained for the identified nodes. Figure 6a,b shows the mode shapes of the tower according to the 35 nodes for both in-plane directions.
- Next, five intended sensors were placed in locations where the mode shapes have maximum linear in-dependency. In other words, the sensors were placed in such a way that the non-diagonal entries of the MAC matrix approach a minimum value [37]. For this purpose, the first sensor was located at the apex of the tower. The next sensor was located at one of the 34 remaining locations to minimize the non-diagonal entries of MAC matrix.
- This procedure was repeated iteratively for the remaining sensors until the best arrangement was found. Eventually, the optimal location for the sensors was obtained as shown in Figure 8c.

## 6. Dynamic Anatomy Identification

### 6.1. Input Excitation

**Figure 9:**
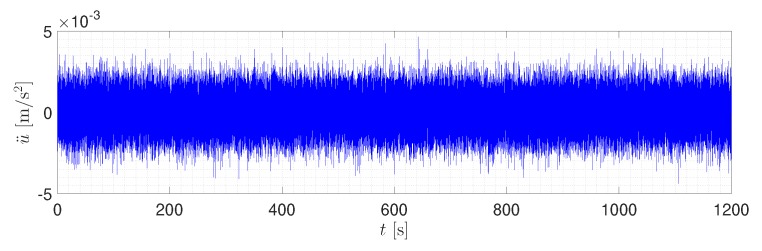
Input excitation signal.

A Gaussian white noise signal was used as an input excitation for the tower. It was a random signal with equal intensity at different frequencies, giving it a constant power spectral density. In other words, a white noise signal had an equal energy at different frequencies [38]. In order to create a white noise signal, the randn function of MATLAB [39] was used. The time step for the input excitation was 0.01 s, and the total time of input signal was 1200 s. It is notable that the input signal was applied to the base of the tower in three perpendicular directions. The main reason for such a long signal was to achieve a stationary condition, which was needed for proper ambient vibration analysis. Figure 9 shows the input signal excitation.

### 6.2. Free Vibration

**Figure 10:**
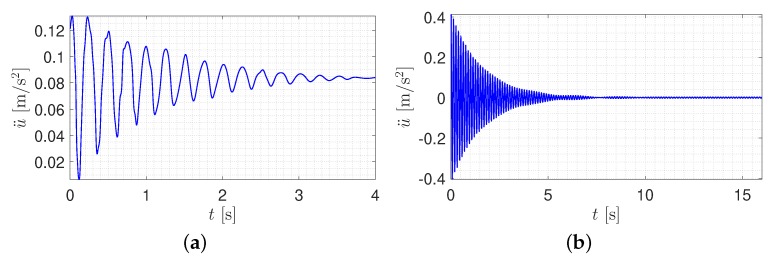
Free vibration response obtained for sensor #5. (**a**) 0–4 s time frame; (**b**) 0–16 s time frame.

Using the random decrement technique (RDT) [40] and establishing the reference degree of freedom (DOF), the correlation between all the DOFs and the reference point was computed. By averaging the generated signal in various time frames, the free vibration of the intended DOF can be identified. It should be noted that the reference DOF represents the most inclusive frequency contents. For instance, Figure 10a,b illustrates the free vibration of sensor #5 in time frames of [0–4]s and [0–16]s, respectively. According to RDT [40], using a larger time window captures more natural frequencies. Here, the sixteen-second time frame represents the first natural mode shape followed by a four-second time frame for the second fundamental natural frequency.

### 6.3. Mode Decomposition

**Figure 11:**
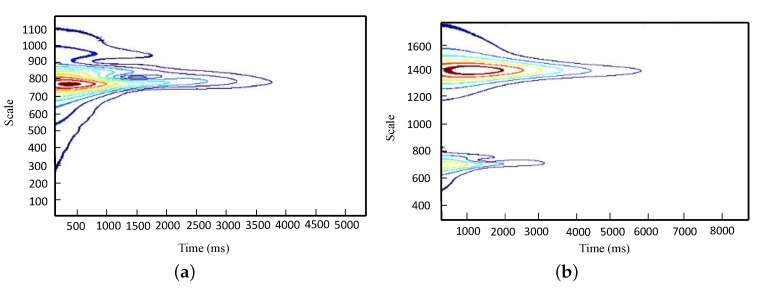
Wavelet coefficient contours. (**a**) Sensor #5; Scale:1,1000; (**b**) Sensor #1; Scale:1,1800.

Execution of CWT on the free vibration obtained in the previous section provides the wavelet coefficient contours in terms of time and scales coefficients. In this section, we detail how a complex Morlet wavelet was utilized with a central frequency of 4.57 Hz. For instance, Figure 11 shows typical diagrams of wavelet coefficients computed for sensors #1 and #5. In order to obtain a free-vibration corresponding to each mode, the 2D plot of summation of wavelet coefficients at their maximum level (peak of the contours) led to the free-vibration of each mode [41].

### 6.4. Natural Frequency and Modal Damping

**Figure 12:**
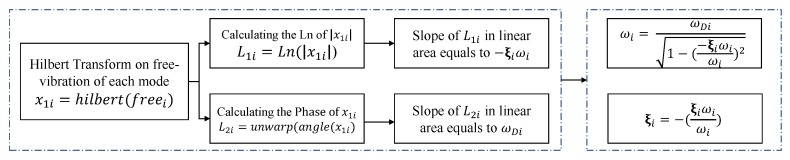
Algorithm to determine $\xi_{i}$ and $\omega_{i}$.

Next, the natural frequency and the corresponding modal damping ratio was computed using the Hilbert transform on the free vibration response of each mode. Figure 12 illustrates an algorithm in order to compute the modal parameters.

1. First, the Hilbert transform of the free vibration corresponding to each mode was calculated.
2. Second, the amplitude and phase of the envelope signal (from previous step) was obtained.
3. Third, the slope of amplitude signal was calculated which leads to $-\xi_{i}\omega_{i}$. Moreover, the slope of the phase signal was obtained which leads to $\omega_{Di}$.
4. Finally, the fundamental of structural dynamics was applied to find $\omega_{i}$ and $\xi_{i}$.

For instance, in order to determine the first and second natural frequencies and the corresponding modal damping ratios (in the lateral direction), one should utilize the scaling of 1400 in wavelet coefficient of sixteen-seconds signal for the first mode, and the scaling of 750 in wavelet coefficient of four-seconds signal for the second mode as shown in Figure 11.

**Figure 13:**
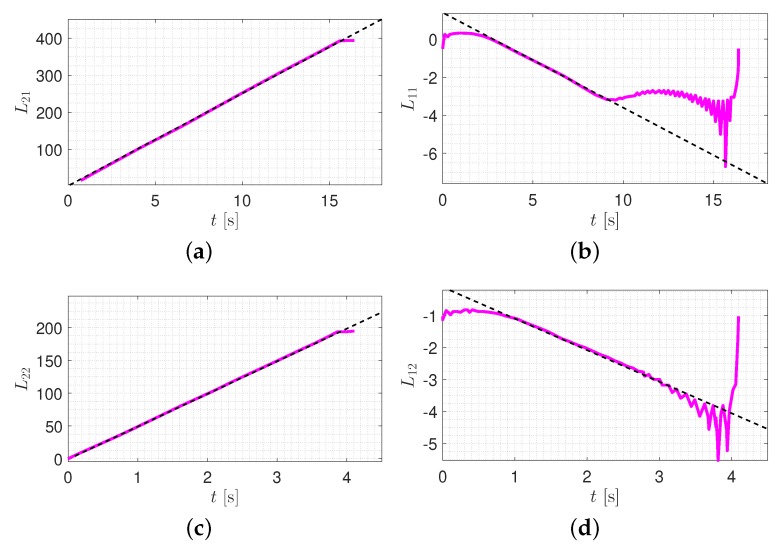
Curve fitting to obtain the modal parameters of first mode, i=1 (first row), and second mode, i=2 (second row). (**a**) $L_{21}$; linear fit: $L_{21}=24.93t+2.089$; (**b**) $L_{11}$; linear fit: $L_{11}=-0.5t+1.4$; (**c**) $L_{22}$; linear fit: $L_{22}=49.76t-0.5955$; (**d**) $L_{12}$; linear fit: $L_{12}=-0.988t-0.1084$.

As it is stated in Figure 12, the slope of $L_{21}$ in the linear region is $\omega_{D1}$. Therefore, according to Figure 13a, $\omega_{D1}$ = 24.93 rad. The slope of $L_{11}$ in the linear region is $-\xi_{1}\omega_{1}$, and according to Figure 13b, the natural frequency and corresponding damping ratio of the first mode (for the case study tower) is obtained with the following simple calculations:

$$-\xi_{1}\omega_{1}=\frac{-0.51}{1}\to \xi_{1}=\frac{0.51}{\omega_{1}}\to \omega_{1}=\sqrt{1-{\left(\frac{0.51}{\omega_{1}}\right)}^{2}}=24.93rad\to f_{1}=\frac{\omega_{1}}{2\pi }=3.968Hz,\;\;\xi_{1}=\frac{0.51}{\omega_{1}}=0.0204$$

The same procedure can be implemented to obtain the second mode properties. According to Figure 12, the slope of $L_{21}$ in the linear region is $\omega_{D2}$. Therefore, according to Figure 13c, $\omega_{D2}$ = 49.76 rad. Furthermore, the slope of $L_{11}$ in the linear region is $-\xi_{2}\omega_{2}$, and according to Figure 13d, the natural frequency and corresponding damping ratio of the second mode is obtained with the following simple calculations:

$$-\xi_{2}\omega_{2}=\frac{-0.9883}{1}\to \xi_{2}=\frac{0.983}{\omega_{2}}\to \omega_{2}=\sqrt{1-{\left(\frac{0.983}{\omega_{2}}\right)}^{2}}=49.76rad\to f_{2}=\frac{\omega_{2}}{2\pi }=7.92Hz,\;\;\xi_{2}=\frac{0.9883}{\omega_{2}}=0.0196$$

In order to obtain other modal parameters of tower (for other direction), a similar approach should be taken. Table 2 presents the first five natural frequencies and modal damping of the tower.

### 6.5. Noise Effect

The information recorded by sensors (i.e., accelerometers) usually contains some level of noise. Signal-to-noise ratio (SNR) is defined as the ratio of signal power to the noise power, and is often expressed in decibels (dB). A ratio higher than 1 (more than 0 dB) indicates more signal than noise [42]:

(7) $$\mathrm{SNR}=20\log\frac{{\mathrm{RMS}}_{\mathrm{signal}}}{{\mathrm{RMS}}_{\mathrm{noise}}}$$

where RMS stands for root-mean-square of a signal.

To simulate a real-world environmental condition, an artificial noise signal should be added to the structural response. In this study, a 10% noise was added to the structural response of tower. This value was at the upper bound value usually used in the literature [43]. Table 3 shows the effect of noise on the structural modal response, and proves that the results are in a desirable range. It should be noted that the fourth mode is the flexural one, which was not identified by the signal processing method.

### 6.6. Mode Shape Identification

In this section, a method proposed by Yang et al. [44] is adapted to identify the structural mode shapes. It can be inferred from previous sections that only one measurement is needed to determine all natural frequencies and the damping ratios. However, to identify mode shapes, the response time histories at all DOFs should be measured. The absolute values of modal element can be determined from the following equation:

(8) $$\frac{\left|\phi_{pi}\right|}{\left|\phi_{qi}\right|}=\exp\left[A_{pi}^{\prime }\left(t_{0}\right)-A_{qi}^{\prime }\left(t_{0}\right)\right]$$

where $\phi_{pi}$ is the value of *i*th mode shape of the structure at the *p*th degree of freedom. Similar interpretation for $\phi_{qi}$ can be sought. The parameters $A_{pi}^{\prime }\left(t_{0}\right)$ and $A_{qi}^{\prime }\left(t_{0}\right)$ are the values obtained from curve fitting to $L_{2i}$ for *p*th and *q*th degrees of freedom at time $t_{0}$, respectively. It should be noted that the value of $t_{0}$ is the time of average value of $L_{2i}$.

The authors of Yang et al. [44] proposed the following relationship to obtain the sign of the mode shape value:

(9) $$\phi_{pi,q}=\theta_{pi}^{\prime }\left(t_{0}\right)-\theta_{qi}^{\prime }\left(t_{0}\right)$$

where $\phi_{pi,q}$ is the difference between the phase value of two signals under evaluation in *p*th and *q*th DOF and *i*th mode. $\theta_{pi}^{\prime }\left(t_{0}\right)$ and $\theta_{qi}^{\prime }$ are also the values obtained from curve fitting to $L_{1i}$ for *p*th and *q*th degree of freedom at time $t_{0}$, respectively. Similarly, the value of $t_{0}$ is the time of average value of $L_{1i}$.

So far, the previous equation determines only the absolute value of mode shapes. In order to obtain the sign of the mode shape entries the following relations are suggested:

(10) $$\begin{array}{cc} \mathrm{if}\;\phi_{pi,q} & =\pm 2m\pi \to \frac{\phi_{pi}}{\phi_{qi}}>0\\ \mathrm{if}\;\phi_{pi,q} & =\pm \left(2m+1\right)\pi \to \frac{\phi_{pi}}{\phi_{qi}}<0 \end{array}$$

**Figure 14:**
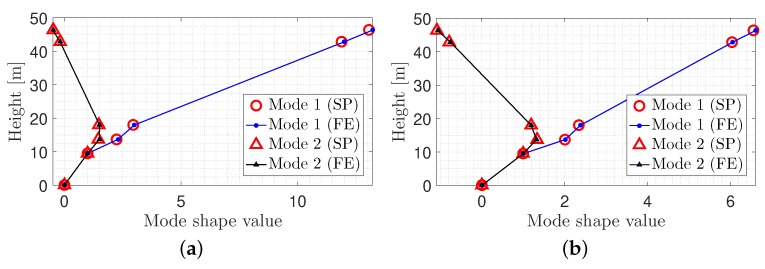
Comparison of the mode shapes obtained from Finite Element (FE) analysis and signal processing. (**a**) Lateral direction; (**b**) Longitudinal direction.

Using the above relations, one can determine the mode shape matrix entries based on free vibration response of those modes with significant participating modal mass. Figure 14 shows the comparison between mode shapes obtained from the finite element analysis and corresponding values from signal processing (SP). It reveals the fact that mode shapes are identified with similar trend.

## 7. Structural Damage Detection

### 7.1. Proposed Damage Detection Procedure

The proposed damage detection procedure is founded on the curvature of mode shapes and the CWT method. First, the curvature of the mode shapes in the intact and damaged structures is calculated using the following relation [45]:

(11) $$\phi_{i}^{{}^{\prime \prime }}=\frac{\phi_{i+1}-2\phi_{i}+\phi_{i-1}}{dh}$$

where $\phi_{i}$ is the value of the mode shape in the *i*th height step, and dh is the corresponding height step (as oppose to time step).

Normally, a large number of data are required to obtain the curvature of mode shapes. However, this study aims to use only five data points associated with the sensor’s output, and subsequently only five mode shape values were available. To overcome this problem, cubic spline function was used in MATLAB [39] to generate sufficient amount of data between these five values. Thereafter, the difference between the curvature of the interpolated mode shapes was computed. Moreover, using CWT on the residual values of the curvature in intact and damaged structure, the location of damage was detected. As it was mentioned in Section 3, Daubechies wavelet was used to monitor the changes occurred in curvature of mode shapes due to the damage.

### 7.2. Damage Scenarios

In order to verify the proposed procedure, four damage scenarios are defined as follows:

Scenario #1; Leg: Stiffness of 12 members of the leg is reduced (in X direction). In order to perception the severity of damage, one can compute the ratio of the damaged element to the total number of elements of the tower. For instance, in this scenario: 12/1978 = 0.61% of the tower’s elements have been damaged,
Scenario #2; Trunk: Stiffness of the top members of the trunk is reduced (in X direction),
Scenario #3; Body: Stiffness of the main elements of the body (at the lower 40% of total height) is reduced (in Y direction), and
Scenario #4; Bridge: Stiffness of the diagonal members of the bridge is reduced (in X direction).

**Figure 15:**
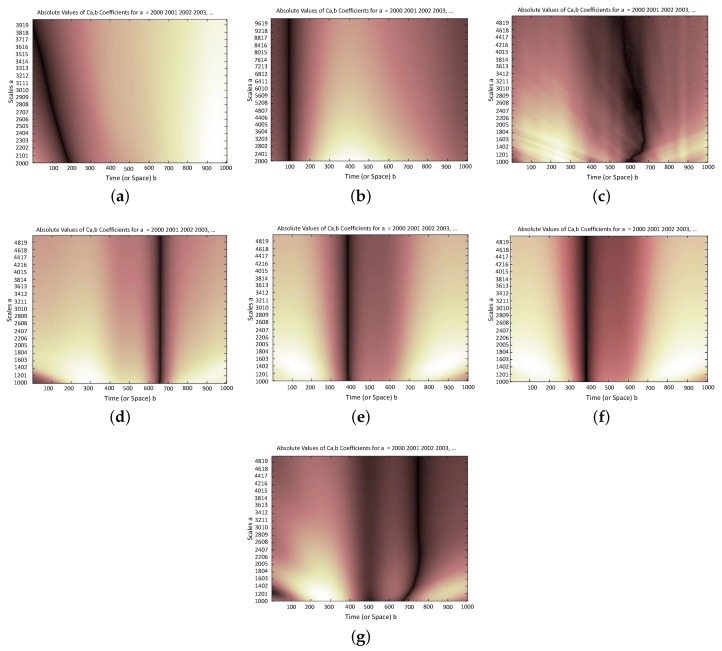
Summation of the wavelet coefficients for different scenarios. (**a**) Scenario #1; 1st mode; X direction; (**b**) Scenario #1; 2nd mode; X direction; (**c**) Scenario #2; 1st mode; X direction; (**d**) Scenario #2; 2nd mode; X direction; (**e**) Scenario #3; 1st mode; Y direction; (**f**) Scenario #3; 2nd mode; Y direction; (**g**) Scenario #4; 2nd mode; X direction.

For all damage scenarios, Figure 15 shows the summation of wavelet coefficients vs. the different scales along the tower height. Results are shown for both 1st and 2nd modes. Moreover, depending on the pre-defined damage elements, the Wavelet coefficients are shown in X or Y directions. The following major conclusions can be drawn:

- Scenario #1: According to Figure 15a,b, at about 10% of tower’s total height, a jump in the summation of wavelet coefficients was observed. This indeed shows the presence of damage. It is notable that the dynamic properties of the upper modes are more sensitive to occurrence of damage. Therefore, as it can be seen in Figure 15a, a variation of the wavelet coefficients of the first mode is not purely vertical, and it is inclined along the height of the structure. Besides, as shown in Figure 15b, variation of the wavelet coefficients of the second mode shape is purely vertical.
- Scenario #2: According to Figure 15c,d, at about 60% of tower’s total height, a jump in the summation of wavelet coefficients was observed due to pre-defined damage. As for the previous scenario, unlike the first mode, a jump in the wavelet coefficients of the second mode is located exactly at the damage location.
- Scenario #3: According to Figure 15e,f, at about 40% of tower’s total height, a jump in the summation of wavelet coefficients was observed due to pre-defined damage.
- Scenario #4: According to Figure 15g, at about 75% of tower’s total height, a jump in the summation of wavelet coefficients was observed due to pre-defined damage. It should be noted that in this scenario, the first mode shape does not contribute to the damage detection process. It is evident that once the upper elements of the tower experience damage, they have a minimal effect on the mode shapes variations.

### 7.3. Comparison with Other Studies

In the method proposed by Xu et al. [2], a total of 22 instruments (including 15 accelerometers and 7 strain gauges) were used for tower damage detection. This method used a discrete damage index for each sensor location. The results of their research showed a good agreement between the actual damage location and the estimated one. A disadvantage of their method is that it relies on a large number of applied instruments. Seyedpoor [46] proposed a two-stage method based on the modal strain energy (MSE) and particle swarm optimization (PSO) to identify the structural damage. This method also had a potential to identify the location and severity of the damage in truss-like structures. Stipulating ideal conditions for obtaining modal parameters and the large number of required sensors are disadvantages of this detection procedure. The method presented by Qu et al. [22] effectively detected the damage that occurred in the major element of the tower. Their method is applicable for the identification of the instability damage only in the major element of the tower. Therefore, the procedure is not suitable for damage detection in a diagonal member of the tower.

As it can be inferred from the results of the present study, the proposed method efficiently detects the damage (both in bottom and top elements). However, it is recommended that in order to make a more reliable decision, this method should be implemented in conjunction with an expert visual inspection. It aims to verify the results obtained through the proposed method by comparing them with physical changes occurred in the structure.

## 8. Conclusions

In this study, a unique technique based on the curvature of the mode shapes along with continuous wavelet transform is presented to detect the potential damage location in the power transmission tower structures. The developed numerical model in this study is based on information provided by the electrical company responsible for the construction of the tower. This numerical model was verified through modal testing of the actual tower. In order to minimize the number of sensors, which eventually leads to a lower cost of the field measurements, a method based on minimizing the non-diagonal entries of the MAC matrix was used. This method led to the usage of only five sensors through out the height of the tower.

The proposed method in this study is based on signal processing of the structure under ambient vibration. In order to simulate the realistic condition of the measured structural response in the field condition, a 10% noise was added to the response of the numerical model. Utilizing the Hilbert–Huang based method, the dynamic characteristics of the tower was identified. The mode shapes were computed based on the method developed by Yang et al. [44]. Four damage scenarios through the tower were defined followed by identifying mode shapes for both the intact and damaged structures.

Finally, by applying CWT to the residual curvature of the intact and damaged structures, the summation of the wavelet coefficient in terms of the scale parameter was determined along the height of the tower. This study showed that the damage scenario #1 to #3 were easily identifiable utilizing the first two mode shapes. However, this was not the case in the scenario #4, which shows the damage using only the second mode shape.

The authors suggest performing further experimental and numerical simulations for towers with different sizes and element configurations. The experimental model could be in the form of small-scale laboratory model to identify the damage location. A sensitivity analysis is also required to understand the accuracy of the damage detection method as a function of number of sensors. This further can be coupled with costs associated with damaged sensors to provide the resiliency of the system.

## Figures and Tables

**Table 1:** Tower’s mode verification results.

Mode	Numerical Model	Experimental Test	Error [%]
First mode (longitudinal direction)	1.04 Hz	1.16 Hz	−10.3
First mode (latitudinal direction)	3.93 Hz	4.08 Hz	−3.7
First flexural mode	5.9 Hz	-	-
Second mode (longitudinal direction)	5.83 Hz	6.01 Hz	−3.0
Second mode (in latitudinal direction)	7.81 Hz	7.98 Hz	−2.1

**Table 2:** Comparison of identified modal parameters with corresponding Finite Element (FE) results.

Mode	Finite Element Model (FEM)		Identified Value	
	Frequency [Hz]	Damping Ratio [%]	Frequency [Hz]	Damping Ratio [%]
1	1.04	2.0	1.07	2.02
2	3.93	2.0	3.97	2.04
3	5.83	2.0	5.91	1.93
5	7.81	2.0	7.92	1.96

**Table 3:** The effect of noise on the identified parameters.

Mode	Without Noise		With Noise	
	Frequency [Hz]	Damping Ratio [%]	Frequency [Hz]	Damping Ratio [%]
1	3.97	2.04	4.02	2.02
2	7.92	1.96	7.98	1.95

## Abbreviations

ABC	Artificial Bee Colony
ANN	Artificial Neural Network
CVT	Capacitive Voltage Transformer
CWT	Continuous Wavelet Transform
DOF	Degree Of Freedom
DOG	Difference of Gaussian
DWT	Discrete Wavelet Transform
FE	Finite Element
FWT	Fast Wavelet Transform
FRF	Frequency Response Function
GA	Genetic Algorithm
IGA	Immune Genetic Algorithm
LS-SVM	Least Square Support Vector Machine
MAC	Modal Assurance Criteria
MK-SVM	Multi-Kernel Support Vector Machine
MSE	Modal Strain Energy
NExT	Natural Excitation Technique
PCA	Principal Component Analysis
PSO	Particle Swarm Optimization
RDT	Random Decrement Technique
RMS	Root Mean Square
SHM	Structural Health Monitoring
SNR	Signal to Noise Ratio
SP	Signal Processing
SWT	Stationary Wavelet Transform
VMD	Variational Mode Decomposition
